# Vacuum Assisted Closure (VAC) at Home: Developing a New Concept

**Published:** 2013-06

**Authors:** Muhammad Ahmad, Humayun Mohmand, Nabila Ahmad

**Affiliations:** 1 Plastic, Reconstructive, Hand and Hair Restorative Surgeon, Islamabad Private Hospital, Islamabad, Pakistan;; 2 Cosmetic Plastic and Hair Restorative Surgeon, Islamabad Cosmetic Surgery Centre, Islamabad, Pakistan;; 3 Foundation University, Islamabad. Pakistan

**Keywords:** Vacuum Assisted Closure, Home, Concept

## Abstract

**BACKGROUND:**

The vacuum assisted closure (VAC) therapy has revolutionized the modern wound care. We developed a new concept of ‘VAC at home’ in order to decrease the hospital cost.

**METHODS:**

The study was conducted in a private hospital from January 2009 to December 2010. Only those patients were included among whom the wounds were not complicated by concomitant injury and the patients were otherwise fit to be discharged from the hospital. The VAC was applied in the hospital. The VAC changes were done after 48-72 hours depending on the general wound conditions. The cost of the VAC changes during the therapy at home was compared with the expected cost of hospital stay.

**RESULTS:**

Thirteen patients were enrolled with a female to male ratio of 1.6:1. The mean age of male patients was 22.4 years as compared to 29.6 years in females. Road traffic accident was the most common cause. Leg (16.2%) was the commonly affected area. The average number of VACs was 15.8. The average cost of the suction machine was 100.1 USD. The average cost of the expected hospital stay during the whole therapy was 68 USD per day. The mean duration of therapy was 35.8 days. The average total cost to be paid to the hospital was 2434.4 USD. Each patient had definitely saved a net of 2739.7 USD.

**CONCLUSION:**

With the increasing experiences of VAC, it can safely be instituted as an ‘outpatient’ procedure to decrease the hospital cost at home.

## INTRODUCTION

The vacuum assisted closure (VAC) therapy (also known as negative pressure wound therapy) since its introduction in 1990’s has revolutionized the modern wound care.^1^^-^^3^ It has been successfully used in the wounds caused by burns, infections, exposed bone or artificial implants and dehiscence.^4^^-^^6^ Negative pressure therapy facilitates the healing by improving the rate of angiogenesis, endothelial proliferation, the integrity of the capillary membrane, capillary blood flow, capillary calibre, and by decreasing interstitial oedema and bacterial burden within the wound.^7^^-^^9^ De Franzo *et al.* described the use of VAC in 75 patients with open, exposed tendons, bones and hardware that otherwise would have required free flap coverage.^2^ There have been few modifications in the VAC system due to the increased cost.^10^^-^^12^

In Pakistan, being a part of developing countries, the people are not able to meet the higher cost of treatment. Majority of the patients undergoing VAC therapy are admitted to the hospital. We developed a new concept of ‘VAC at home’ in order to decrease the hospital cost.

## MATERIALS AND METHODS

The study was conducted in a private hospital from January 2009 to December 2010. The VAC therapy was used in 13 patients having open wound. All these wounds failed to be closed primarily or required flap coverage. Only those patients were included among them, the wounds were not complicated by concomitant injury and the patients were otherwise fitted to be discharged from the hospital. The decision was done on case to case basis. The VAC was applied in the hospital. The VAC changes were done after 48-72 hours depending on the general wound conditions. The size of the wound was noted before the start of VAC and at the end of the therapy. 

**Fig. 1:**
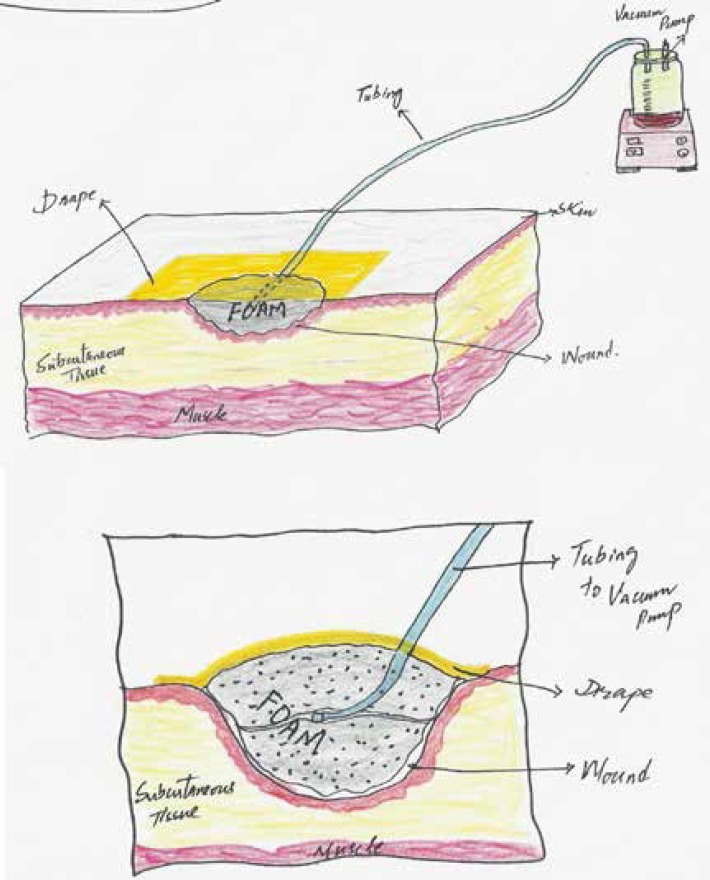
VAC application

The VAC consisted of a double layer of ½ inch thick foam. A drain no. 16 or 18 G was used. The tube was connected to the connecting tube of the suction machine by a 5 ml disposable syringe (Figure 1). The foam and part of the drain was covered with pre-op drape (Opsite®) extending about 3-5 cm beyond the foam margins to create an airtight seal. The suction machine was set at a pressure of 100-125 mmHg with 20 minutes ‘ON’ and 40 minutes ‘OFF’ cycles. The cycles started from 6 AM and continued till 12 midnight (almost 18 hours). The VAC dressings were changed after 48-72 hours. Patients were advised to report immediately if there was any leakage or excessive exudate/bleeding in the drain tube.

At the end of the procedure, wound dimensions were noted. The patients underwent definitive procedure. The cost of the VAC changes during the therapy at home was calculated and compared with the cost of the expected cost of hospital stay. 

## RESULTS

Thirteen patients were included in the study with a female to male ration of 1.6:1. The mean age in male patients was 22.4 years (range of 7–59 years) as compared to 29.6 years in females (range of 15–45 years). Road traffic accident was the most common cause (Table 1). Leg (16.2%) was the commonly affected area. The average size of the wound at the start of the therapy was 5.9×11.5 cm and 4.5×9.2 cm at the end of the therapy. The average number of VACs was 15.8. The average cost of the suction machine was 100.1 USD (range of 87–101 USD). The average cost of the expected hospital stay during the whole therapy was 68 USD per day. The mean duration of the therapy was 35.8 days (range of 28–49 days). The average total cost to be paid to the hospital would have been 2749.55 USD (range of 1883.7–4558.1 USD). Each patient had definitely saved a net of 2612.61 USD (Table 2).

**Table 1:** Characteristics of the Patients

**Case No.**	**Age** **(years)**	**Sex**	**Cause**	**Area**	**Size ** **(at the start)**	**Size** **(at the end)**	**Therapy duration**	**No. of VACs**	**Co-morbid condition**
1	7	M	Road-traffic accident	Right heel and sole	5×10	3×8	45	22	-
2	21	F	Road-traffic accident	Left heel	6×12	4×10	31	10	-
3	15	M	Road-traffic accident	Right leg	6×28	4×21	35	16	-
4	28	M	Burn	Right hand dorsum	4×4	3×3	27	13	Smoker
5	36	F	Workplace accident	Left leg	4×10	3×9	36	19	Diabetic
6	15	F	Firearm injury	Right anterior tibia	6×7	4×5	41	20	-
7	19	M	Road-traffic accident	Dorsum of foot	7×11	6×9	38	17	-
8	31	F	Infection	Left forearm	9×13	7×11	35	16	Smoker, diabetic
9	17	M	Diabetic ulcer	Left hand dorsum	5×6	4×4	29	10	Diabetic
10	50	M	Road-traffic accident	Right leg fasciotomy	7×21	5×19	28	10	-
11	45	F	Road-traffic accident	Left anterior tibia	5×7	4×4	33	16	-
12	59	M	Bed sores	Back	5×7	4×6	39	15	Diabetic smoker
13	34	M	Burn	Left foot dorsum	8×13	7×11	49	21	Smoker

**Table 2:** Comparison of the cost

**A. Cost of hospital stay**	
Mean duration of VAC therapy	35.8 days
Estimated total cost of hospital stay	35744.19 USD
Estimated total coast of hospital stay per patient	2749.55 USD
B. VAC at home	
Total machine cost and electricity bills	1780.23 USD
Total machine cost and electricity bill per patient	136.94 USD
Difference (saving) if VAC at home used	
Total (A–B)	33963.96 USD
Per patient (A–B)	2612.61 USD

Currency conversion rate 1 USD=86 PKR

**Fig. 2:**
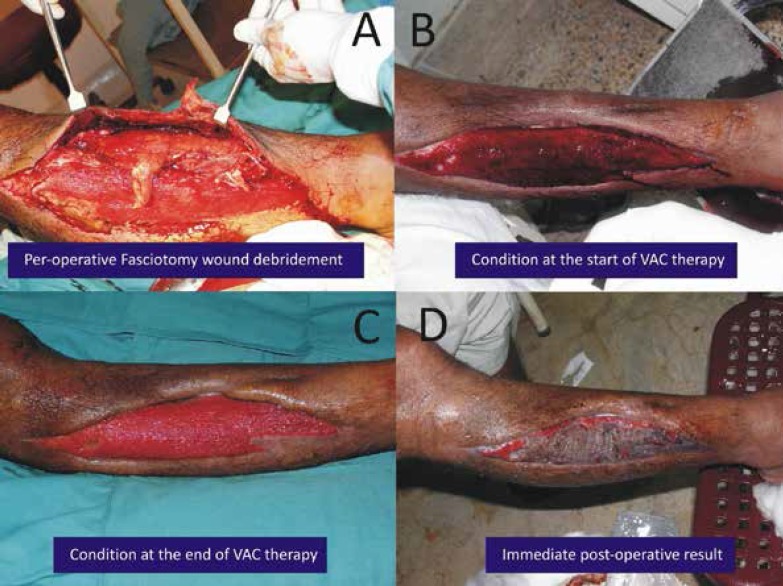
Preoperative and postoperative results of fasciotomy wound

Case one was a 50 years old male patient suffered a road traffic accident. He was managed conservatively by orthopaedic surgeon and later developed compartment syndrome. He underwent fasciotomy and was later handed over for further management. The patient underwent extensive debridement and later VAC was started. The wound healed satisfactorily and later skin grafting was performed (Figure 2).

**Fig. 3:**
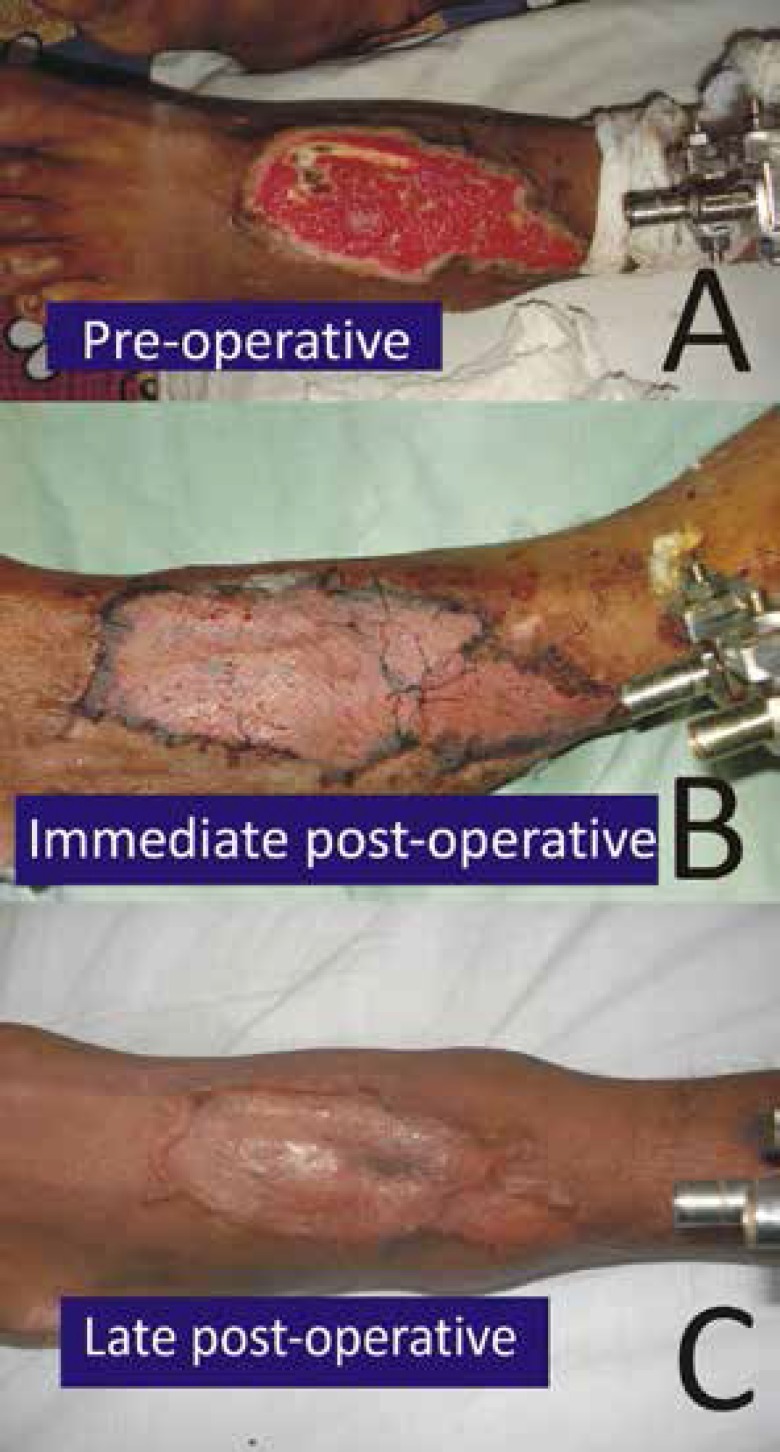
Preoperative and postoperative results among male patients with wound on dorsum of foot

Case two wa a 19 years old male who had road traffic accident resulting in a wound on dorsum of right foot and tibial fracture. The fracture was fixed by orthopaedic surgeon and VAC was started for wound. The wound was later covered with skin graft (Figure 3).

## DISCUSSION

The use of VAC is increasing especially in reconstruction of complex wound defects. The VAC acts as a new step in the ‘reconstruction ladder’. The VAC enhances the tissue granulation which makes it possible to use less complex reconstruction options, e.g., converting the wounds acceptable for the skin grafting which otherwise would have required flap coverage.^10^ With the increasing use of VAC, the patients’ morbidity is reduced and in most of the studies, the patients needed to be admitted in the hospital which definitely adds to the financial burden on the patients. In areas where the concepts of health insurance are not well-established, the extra hospital cost could be a deciding factor. If the VAC therapy can be continued at home, it would decrease the hospital cost.

In the present study, the cost of the local suction machines was mush less than the cost of expected hospital stay if the therapy would have been continued as ‘in-patient’. In the present study, the average duration of VAC therapy was 32.69 days (range of 27-49 days). The estimated cost of hospital stay was 68USD per day (2749.55 USD per patient). Whereas the average cost of the local suction machine was 100.1 USD and the estimated average electricity bill was 47.7 USD. Each patient undergoing VAC at home in the current situation spent 147.8 USD saving more than 2000USD if he had to be admitted in the hospital during the whole therapy. We did not calculate the cost of the materials used in the treatment as it were to be used in any case.

In a similar study by Kolios *et al.*, the average VAC cost per patient was 3266.39 USD which is much less than the cost of VAC if used at home (147.8 USD per patient).^13^ The high cost of medical care has come under scrutiny by payers and physicians.^14^ In another study by Nather *et al.*, the average cost of VAC therapy was 2330 $ per day which is again more than 15% higher as compared to the average cost in the present study.^15^ The use of locally available open cell foam (autoclaved before use), the local vacuum machine, all helped to reduce the cost in our setup.

Vacuum assisted closure therapy is very helpful in the treatment of wide variety of conditions. With the increasing experiences of VAC, it can safely be instituted as ‘outpatient’ procedure to decrease the hospital cost at home.

## CONFLICT OF INTEREST

The authors declare no conflict of interest.

## Footenote

Please cite this paper as:

Ahmad M, Mohmand H, Ahmad N. Vacuum Assisted Closure (VAC) at Home: Developing a New Concept. World J Plast Surg 2013;2(2): 87-92.
